# Reaction Factoring and Bipartite Update Graphs Accelerate the Gillespie Algorithm for Large-Scale Biochemical Systems

**DOI:** 10.1371/journal.pone.0008125

**Published:** 2010-01-06

**Authors:** Sagar Indurkhya, Jacob Beal

**Affiliations:** 1 Computer Science and Artificial Intelligence Laboratory, Massachusetts Institute of Technology, Cambridge, Massachusetts, United States of America; 2 BBN Technologies, Cambridge, Massachusetts, United States of America; Center for Genomic Regulation, Spain

## Abstract

ODE simulations of chemical systems perform poorly when some of the species have extremely low concentrations. Stochastic simulation methods, which can handle this case, have been impractical for large systems due to computational complexity. We observe, however, that when modeling complex biological systems: (1) a small number of reactions tend to occur a disproportionately large percentage of the time, and (2) a small number of species tend to participate in a disproportionately large percentage of reactions. We exploit these properties in LOLCAT Method, a new implementation of the Gillespie Algorithm. First, factoring reaction propensities allows many propensities dependent on a single species to be updated in a single operation. Second, representing dependencies between reactions with a bipartite graph of reactions and species requires only 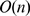 storage for 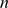 reactions, rather than the 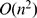 required for a graph that includes only reactions. Together, these improvements allow our implementation of LOLCAT Method to execute orders of magnitude faster than currently existing Gillespie Algorithm variants when simulating several yeast MAPK cascade models.

## Introduction

Dynamic Monte Carlo methods are a common means of simulating the time-evolution of chemical systems. The Gillespie Algorithm (SSA) [1] is the standard algorithm for this process, and has inspired a variety of derivative methods that speed up computation, including the Optimized Direct Method (ODM) [2] and the Next Reaction Method (NRM) [3]. These methods, however, are still computationally costly. Speeding up the Gillespie Algorithm and related hybrid methods will likely play an important role in advancing the productivity of computational systems biology.

In this paper, we develop a new algorithm, LOLCAT Method, that can speed up the exact stochastic simulation of a large class of well-mixed chemical systems by orders of magnitude. The ability to perform simulations orders of magnitude faster will allow scientists to revisit problems that were previously computationally intractable, such as whole-cell simulation. Other applications include the simulation of large genetic regulatory networks and metabolic pathways. Further, we demonstrate that this algorithm can operate on a typical desktop personal computer, bringing the simulation of extremely large chemical systems into the reach of the general scientific community. Our implementation of LOLCAT Method is publicly available at [4]

### Simulating Chemical Systems

There are two main approaches to simulating the time-evolution of chemical systems: deterministic and stochastic. Deterministic methods express chemical concentrations with real numbers, and evolve concentrations forward in time via differential equations. Stochastic methods, on the other hand, express concentrations as non-negative integers, and evolve them in discrete steps: at each step, a reaction is chosen and executed, transforming a set of reactants into a set of products and advancing the simulation time by a small amount. The reaction and time step are chosen randomly according to a distribution that produces a statistically correct simulation of the chemical system.

Stochastic methods are generally much slower because they simulate every chemical interaction explicitly, but produce guaranteed valid results for systems where concentrations are very small. Deterministic methods are much faster, but do not handle small concentrations well. This is particularly problematic when dealing with models of biological systems, since these often contain important chemical species with dozens of molecules or less.

Hybrid methods attempt to resolve this dilemma by mixing deterministic and stochastic methods, and are sped up significantly by techniques such as tau-leaping at the expense of not being exact (although they are becoming increasingly accurate and are good enough for many problems) [5], [6]. These hybrid methods are often based on some exact method [7], so LOLCAT Method can likely increase the speed of even these hybrid methods. For a thorough treatment of stochastic versus deterministic simulations, see [3].

### Stochastic Simulation Algorithm

The Stochastic Simulation Algorithm (SSA), also known as the Gillespie Algorithm, is a standard, well established approach for computing statistically correct trajectories of the time evolution of spatially homogeneous chemical systems. Unfortunately the SSA is extremely computationally expensive. We review the SSA briefly to build a foundation for deriving LOLCAT Method.

Consider a chemical system of constant volume (for systems with variable volume, see the Supporting Information S1) in which there is a set of species 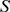 and a set of reactions 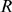 governing the interactions of these species. Assume that all reactions have at most two reactants and two products (any reaction with more than two reactants or products can be factored into reactions with at most two reactants and products), as well as a constant reaction rate. The SSA is then as follows:

1. Initialize simulation time 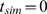, and the initial concentration of each species.
2. For each reaction 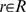, compute the reaction propensity 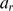; if reaction 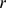 is 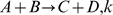 (where 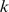 is the reaction rate) we have 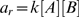, where 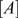 and 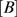 are the number of molecules present in the system for species 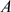 and 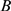 respectively. Note that if 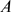 and 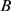 are the same species, then we instead have 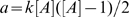, and for reactions with less then two reactants and products, we use the the chemical 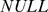, which is omnipresent with constant concentration 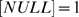. Compute the sum of the propensities: 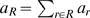.
3. Choose a reaction, 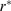, at random from the weighted distribution of reaction propensities. Each reaction 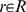 has probability 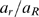 of being chosen.
4. Execute reaction 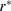: subtract 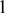 from the concentration of each reactant, and add 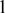 to the concentration of each product.
5. Update the simulation time: 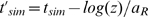, where 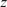 is chosen uniformly randomly from 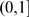

6. Record species concentrations of interest as desired for the experiment. Go to step 2 until the simulation is completed (usually when some desired amount of time has been simulated, or when a desired number of iterations have been completed).

Each iteration of the SSA may also be viewed as two phases: a read phase, in which the algorithm chooses a random number and maps it to the reaction to execute (step 3), and a write phase, in which the reaction is executed, propensities are adjusted, and simulation time is advanced (steps 2, 4, and 5). We use this two-phase view in describing LOLCAT Method, as focuses attention on the interaction of the algorithm with its supporting data structures.

### Review of Prior Methods

The SSA has inspired a variety of derivative methods that speed up computation. The two most significant are the Optimized Direct Method (ODM) and the Next Reaction Method (NRM).

The Optimized Direct Method (ODM) [2] stores the reactions in an array. First, a random number 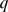 is chosen uniformly from the interval 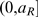. The algorithm then steps through the array (linearly) subtracting 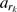, (where 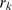 is the 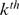 reaction) from 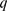 until 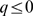, and at that point takes 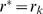. Several simulations are run ahead of time to determine an average propensity for each reaction and the reactions are sorted by average propensity from greatest to least. In most biochemical systems, a relatively small number of reactions occur a disproportionately high percentage of the time, so ODM's linear search allows for these reactions to be quickly detected at the front of the array. The time for choosing a reaction is 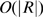, but the sorting causes it to be much faster on average.

To recompute the propensities, ODM uses an update dependency graph (UDG) that maps each reaction 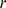 to a list of 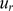 reactions whose propensities should be updated upon execution of 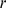. As a result, recomputing propensities takes 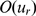 time. For large systems, 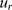 is often high for the most frequently occurring reactions, dramatically slowing down ODM.

The Next Reaction Method (NRM) [3] takes a different approach than ODM. NRM computes for each reaction the amount of time before it will next occur. It then stores the reactions in a binary min-heap structure, so that the next reaction to occur is always at the top. This reaction is executed, and then the necessary reactions have their propensity (and wait-times) updated (using a UDG as ODM does). However, as each reaction propensity is updated, it must be shifted up or down in the heap to maintain the min-heap property. While choosing a reaction takes 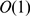 time, execution and update requires 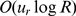 time. Thus, for large systems the size of 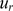 tends to slow NRM dramatically, just as it does ODM.

Other methods exist, but their performance and algorithmic structure are similar to ODM or NRM. For example, the Sorting Direct Method [8] is like ODM with dynamic reordering of reactions as propensities change, and Logarithmic Direct Method [9] is like ODM with propensities are stored in a binary structure for faster lookup of 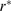. Although these methods sometimes perform better, the measured speedup is only a small constant, and we thus compare only against ODM and NRM.

## Methods

We make two observations that appear to apply in many models of large scale biochemical systems:

- A small number of reactions tend to occur a disproportionately large percentage of the time.
- A relatively small proportion of the species take part in a relatively large proportion of the reactions (e.g. ATP and water in some cellular systems). One consequence is that these change concentration with a disproportionately high frequency. We will refer to these species as super-species.

LOLCAT Method exploits these observations in two ways. First, when possible, reactions are grouped by a common reactant and the common reactant's concentration factored out, allowing the simultaneous update of the propensities of reactions in that group. Second, the update dependency graph is stored in a bipartite representation to reduce the amount of computer memory required. We discuss these ideas in detail one at a time, introducing necessary data structures along the way, and then formally describe the algorithm in its whole.

### Factoring Out Common Reactants

The propensities of reactions with one or more common reactants can be grouped together:

1. The sum of the propensities for a set of 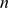 reactions 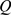 with *a single common reactant* 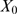 can be factored as: 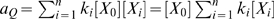, where 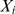 is the other reactant in the 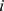
th reaction in 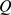 and 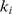 is its reaction rate.
2. The sum of the propensities for a set of reactions 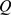 with *both reactants in common* can be factored as: 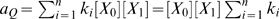.

From this observation, we derive a brief mathematical foundation which we will employ in later developing the data structures for LOLCAT Method. Consider a set of reactions 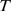 in which all reactions have a common reactant 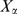. We may then further partition this set of reactions into sets 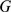 and 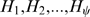, where each set of reactions 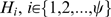 has a unique secondary common reactant 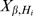, and 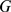 contains all other reactions in 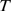 (those without shared secondary common reactants, or where the number of reactions sharing a secondary common reactant is too small for a second factoring to be beneficial).

The sum of the propensities of the reactions in 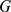 is
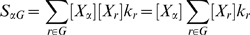
(1)

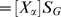
(2)defining 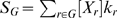. Notice that we factored out the 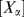 common to all reactions in 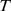.

The sum of the propensities of the reactions in 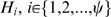 is
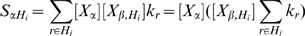
(3)

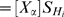
(4)defining 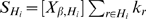. Note that we factored out the common reactant 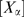 from all 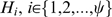, and factored out each secondary common reactant 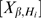 as well from each 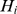. Also note that 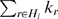 is a constant.

We let 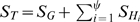, and 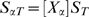. 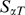 is the sum of the reaction propensities for all reactions in T. Now we show some operations we can perform.

1. If we increment the concentration 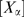 by some amount 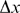, then we can produce updated values as follows:
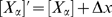
(5)

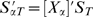
(6)Without the use of factoring, this would have incurred 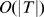 operations rather than 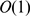. This provides one of the main speedups of LOLCAT Method.
2. If we increment 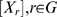 by 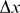, then we have:
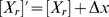
(7)

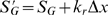
(8)

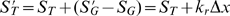
(9)

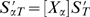
(10)
3. If we increment 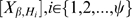 by 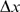, then we have (noting again that 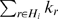 is a constant):
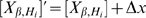
(11)

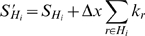
(12)

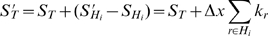
(13)

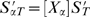
(14)

The latter two provide less speed-up, but factoring still provides some benefit.

### Reaction Group Data Structures

LOLCAT Method embodies these mathematical insights in a specialized data structure that we call a *cloud*. Each cloud stores the factored propensities for a group of reactions sharing a common species. Continuing with our sets 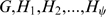 and 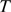 used previously, we define the cloud's *factor-species* as their common reactant 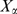. The cloud then consists of a *primary tree*, a set of *sub-trees*, and a slot which holds 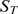.

The *primary tree* is a balanced 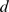-ary tree with each leaf node having a one-to-one mapping to a reaction in 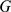. The value of the leaf node tied to reaction 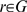 is 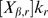 (where 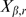 is the reactant in 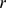 that is not the *factor-species* 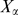), and the value of any non-leaf node holds the sum of its child nodes. Thus the root of the primary tree holds the value 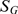.

Note that we choose 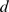-ary trees rather than binary trees. The branching factor of a tree controls its height: shorter trees are faster to update, but more branches require more tests to find a node. On modern processors, the optimal branching factor may be much greater than the 2 dictated by binary trees. See the Supporting Information S1 for details.

The cloud also has 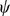 different *sub-trees*, which are also 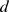-ary trees. Each leaf node of the 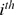 sub-tree has a one-to-one mapping to a reaction in 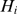: the value of the leaf node tied to reaction 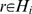 is 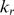. Each non-leaf node holds the sum of its child nodes. Thus the root node of the 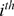 sub-tree holds the value 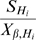.

In our implementation of LOLCAT Method, clouds are constructed by a preprocessing program that takes in the input system to be simulated, along with samples of the average propensities of each reaction over trial simulation runs. Reactions are assigned to clouds using a greedy approach:

1. A potential cloud is created for each species in the system, and each reaction is put into any cloud that could hypothetically contain it.
2. The sum of propensities of reactions in each cloud (based on a trial run) is computed. This heuristic score estimates how often the cloud's grouping will be taken advantage of.
3. The cloud with the best score is fixed, and all reactions in the newly fixed cloud are removed from any other clouds they are in.
4. Rescore each cloud (discarding empty clouds) and continue the process until every reaction is part of a fixed cloud.

Within a cloud, reactions with two common reactants are assigned to the primary tree unless there are enough of them (three or more in our implementation) that creating a sub-tree for them is deemed worthwhile.

Clouds are stored in a balanced 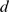-ary tree, which we call the *main tree*, for fast access. Each leaf node has a unique one-to-one mapping with a cloud and holds the value 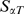 for that cloud. Each non-leaf node holds the sum of its child nodes, such that the root node of the tree holds the sum of the propensities of all reactions that reside in a cloud. When selecting a reaction using a random number 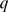, we traverse down this tree using our random number to determine which child node to proceed to until we reach a cloud. If there are 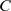 clouds, then this operation takes 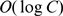 time. Once we reach a cloud we rescale the random number to aid in selecting a reaction from the cloud. See the formal description of the LOLCAT Method below for more details.

Finally, to take advantage of the observation that a few particular reactions will occur disproportionately frequently, we segregate these reactions out and store them in a small static array, which we call the *super-cache*. This holds the reactions with the largest average propensity (and therefore the reactions most likely to be executed during the simulation). We also maintain a sum, 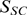, of the propensities in the super-cache. The super-cache is always searched first, and its size is chosen to balance the cost of linear search against the advantages of local access.

**Figure 1:**
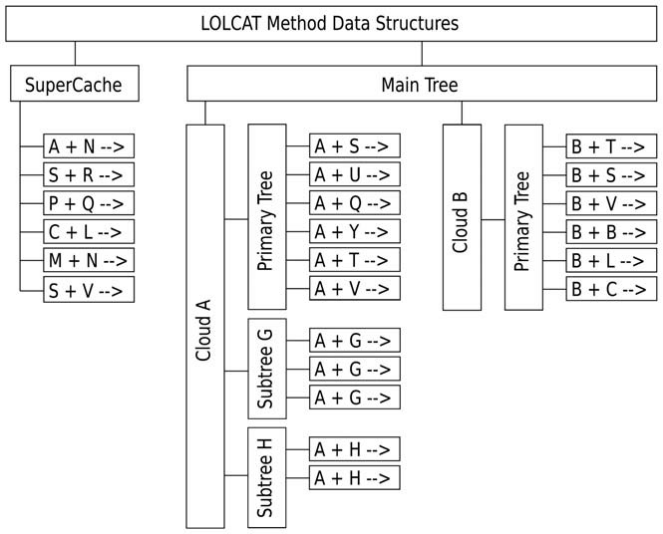
Example of LOLCAT Method's data structure. In practice, our implementation only creates subtrees for sets of reactions significantly larger than those shown in Cloud A's subtrees, for efficiency reasons. As a result, most clouds are like Cloud B and have no subtrees.

Figure 1 shows an example of reactions organized by LOLCAT Method into a super-cache, main tree, and clouds.

### Using a Bipartite Update Dependency Graph

After a reaction executes, the reaction propensity must be updated for all reactions whose reactants' concentrations have changed. ODM and NRM store the dependencies between reactions in an update dependency graph (UDG) where each node is a reaction and directed edges connect it to all reactions whose propensities must be updated when it executes. When the same species appears in many reactions as a reactant but not a product, those reactions form a clique, with every reaction pointing to every other reaction. For a system of 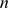 reactions, the number of edges is 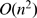. Because it is often the case in large scale biochemical systems that some species are involved in a relatively large proportion of the reactions, such systems may often approach this bound.

LOLCAT Method uses a bipartite UDG instead, where each reaction points to the species whose concentrations it changes, and each species points to the reactions in which it is a reactant. Each reaction has two reactants and can affect the concentration of no more than 4 species, so the number of edges is bounded above by 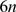.

The difference between 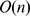 and 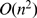 is not noticeable for low 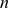, but as 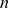 grows the amount of memory required to store the graph grows. A reaction-only UDG bounded by 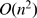 rapidly overwhelms the cache and eventually even the main memory (RAM), making it far slower or even impossible to execute. Thus for large systems where some species participate in many reactions there is no question that we should use a bipartite UDG. To plainly demonstrate this point, we have created and benchmarked (see below) a Modified ODM (MODM) that is identical to ODM except that it uses a bipartite UDG.

Note that every time a reaction is executed, the set of update computations that must be performed are identical. An additional minor speed increase may thus be obtained by compiling this dependency graph together with the cloud structures into a cache of per-reaction and per-species update instructions rather than using a generic function that references the graph. See Supporting Information S1 on the Optimized Interpreter and Figure S1 for details.

### Formal Description of the LOLCAT Method

The following algorithm is repeated until a termination criteria is satisfied, such as completing a desired number of iterations or 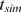 reaching some desired value:

1. **Phase 1: Choose a reaction to execute**
  - Generate a random number 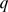 uniformly in the interval 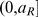.
  - If 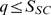, then 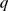 maps to the super-cache. If it does, step linearly through the super-cache, subtracting the propensity of each reaction until 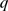 is less than or equal to the propensity of next reaction. Let this reaction be 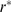, and go to **Phase 2**.
  - Otherwise, subtract 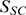 from 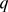 and descend down the main tree. At each level, subtract the propensity of left branches from 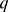 until the propensity of a branch is greater than 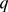: this is the branch that is selected. When this is a leaf, it contains 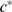, the cloud that 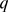 maps to. Let 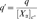.
  - If 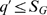 then 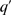 maps to 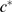
's primary tree. If it does, then descend down that primary tree as above, subtracting the cumulative propensity of untaken left branches from 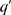, to find which reaction in the primary tree 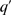 maps to. Let this reaction be 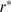, and go to **Phase 2**.
  - Otherwise, step through the list of sub-trees of 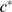, subtracting the 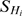 of each non-selected sub-tree from 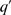. The sub-tree for which 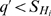 is the sub-tree that 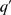 maps to. Let 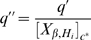.
  - Descend down the sub-tree 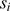 as above, subtracting the cumulative propensity of untaken left branches from 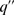 to find the reaction that 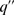 maps to. Let this reaction be 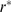, and go to **Phase 2**.
2. **Phase 2: Execute the chosen reaction**
  - Update the simulation time: 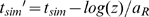, where 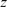 is a random number in 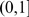.
  - Use the (compiled) update graph to adjust species concentrations and reaction propensities for an execution of 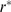, as per the mathematical rules given above. For details on the efficient implementation of updates and recording, see the Supporting Information S1 on the Optimized Interpreter and Figure S1.

Note that the actual propensity of each individual reaction (and thus its probability of selection) is precisely identical to that used by the Gillespie Algorithm—LOLCAT Method only organizes and records this information differently. Because the probability of selecting any given reaction and the effect on propensities following its selection is identical for LOLCAT Method and the Gillespie Algorithm, we are guaranteed that LOLCAT Method is a correct implementation of the Gillespie Algorithm, assuming that a sufficient number of bits are used in the floating point representations of propensities.

## Results

We experimentally verified the speed advantage of LOLCAT Method on a set of yeast MAPK cascade models obtained from the Yeast Pheromone Model repository [10]. Note that we are concerned only with the fact that these are complex biochemical models that a scientist would reasonably wish to simulate, not with the correctness of these particular models. Six different versions of the cascade model were used, each with a different number of reactions and species. Each model was run to steady-state for 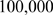 seconds (about 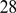 hours) of simulation time. We then changed the pheromone concentration from 0 nM to 100 nM for each model, and benchmarked ODM, NRM, MODM and LOLCAT Method.

For the purpose of benchmarking the various algorithms, all simulations were written in ISO compliant C++ and carefully optimized. The Intel C++ Optimizing Compiler v. 10.1 was used to compile the source to machine code. Benchmarks were measured on a machine with an Intel Xeon 5355 Quad-Core 2.66 Ghz 64-bit processor with the SSSE3 instruction set, a 4 MB cache, 8 GBs of RAM, 12 GB swap space and a 250 GB hard drive. Our implementation of LOLCAT Method [4] is publicly available in MIT's DSpace archival storage at http://hdl.handle.net/1721.1/46710.

**Figure 2:**
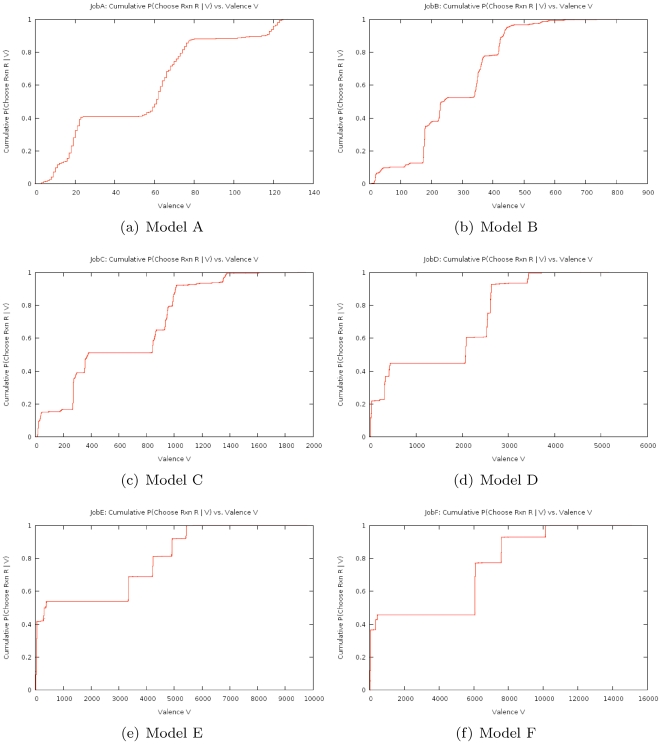
Cumulative distribution function (CDF) plots of reaction valence for all six models. To compute the CDF we first computed the PDF of reaction valence, weighting each reaction valence by the reaction's average propensity over a pre-computed sample trajectory. We then computed the CDF from the PDF to increase visual salience of the sparsely distributed weights. A steep climb near a particular valence means a significant probability of a randomly chosen reaction having that approximate valence. This, in turn, often indicates the presence of a super-species that is involved in many reactions and benefits greatly from factoring.

For each model, we ran 10 trials of 40 million iterations for each of the four different algorithms and recorded the mean and standard deviation of the runtimes. We computed the ratio of the runtimes, normalizing by the mean time taken by LOLCAT Method, and present the resulting speedup factors in Table 1. We do not report the preprocessing time for the various methods, as preprocessing needs to be done only once for a batch of many simulations (often thousands or more per batch) and all of the methods evaluated completed preprocessing in less than one second. As the structure of the reaction graph is critical to the behavior of simulations, we also computed the distributions of the reaction valences (the number of other reactions whose propensities change when the reaction executes) with respect to propensity in the 6 different models after they had reached steady-state and the pheromone was added, and present the cumulative distribution function for all model in Figure 2.

**Table 1:** Benchmarking results for ODM, NRM, MODM and LOLCAT Method.

System Parameters Runtimes(s) 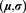
ID	# Rxns.	# Species	ODM	NRM	MODM	LOLCAT
A	2040	236	8.45, 0.04	25.52, 5.75	25.08, 0.12	3.06, 0.14
B	11492	5092	61.08, 0.17	162.68, 6.15	64.21, 0.22	1.68, 0.04
C	35003	11402	152.18, 0.64	374.73, 4.63	166.06, 0.46	3.73, 0.06
D	84301	15087	374.66, 2.30	720.35, 62.32	390.85, 1.41	4.73, 0.13
E	162150	14766	640.72, 3.01	964.49, 99.97	623.39, 1.58	1.88, 0.04
F	292190	15287	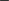	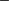	2527.93, 61.09	9.30, 0.09

Six versions of the Yeast Pheromone Model [10] were benchmarked. Slowdown factors are mean time normalized against the performance of LOLCAT Method for each model. The “
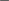
” in the last entry for ODM and NRM indicates that those simulations could not be run because the dependency graph consumed more than the RAM and swap space, roughly 20 GB, of the host machine. The varying structure of the simulated system may account for the non-uniform scaling of runtimes.

In every case, LOLCAT method greatly outperforms the other methods. As the size of the model increases, the advantage of LOLCAT rises by orders of magnitude. Indeed, ODM and NRM were not even able to run the largest model we benchmarked due to the size of their dependency graph, while Model D consumed more than 4 GB of RAM and Model E consumed more than 12 GB of memory. This means that ODM and NRM require a 64 bit architecture to run models D, E and F, while LOLCAT method consumed less than 4 megabytes and fit into the L3 cache, and can run on a 32 bit architecture if desired.

## Discussion

LOLCAT Method uses two key ideas: (1) grouping reactions with common reactants and updating the propensities of many reactions in a single operation, and (2) using a bipartite update dependency graph of species and reactions, resulting in a much more compact form. Note that the factoring of reactions allows for the dependency graph to be further compressed beyond the simple species-based dependency graph used by MODM. These two principles allow LOLCAT Method to outperform other popular methods by orders of magnitude on the chemical systems we benchmarked. Furthermore, the performance advantage of LOLCAT Method is expected to increase as the size of the systems being modeled increases. LOLCAT Method is also able to gracefully handle systems with a large number of interdependent reaction propensities, something that all previous methods are not able to do.

It is important however, to note that the speedup of LOLCAT Method cannot be measured simply as a function of the number of reactions or species or even the average reaction valence. Rather, the speedup factor seems to be best measured by studying the reaction valence CDF of the system being modeled. However, we are optimistic that many large scale biochemical systems will prove to have CDFs that mean they are amenable to orders of magnitude speedup via LOLCAT Method simulation.

Note also that the results presented above do not separate the advantage due to factoring from the advantage due to logarithmic search for a reaction to execute, but we believe the first to be dominant for large systems due to the large size of clouds generated by the greedy search in our application of LOLCAT to these models.

The authors would like to acknowledge the recent publication of another variant of the Gillespie Algorithm, SSA-CR, published in which Phase 1 of the algorithm is reduced to 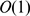 time via a clever method based on rejection-sampling [11]. Phase 2 of this algorithm, however, still cannot scale well when the average reaction valence is high, and in the case of high reaction valence systems, Phase 2 dominates the cost of the Gillespie Algorithm. Thus, we believe that if we were to benchmark SSA-CR, LOLCAT Method would outperform it by a similar margin.

We would also like to note that there is an aggressive trend in computational biology to tackle computationally expensive problems by throwing hardware at the problem. Sometimes this approach generates interesting methods, such as the use of FPGAs [12]. Some of these gains can be realized on desktop computers simply by paying careful attention to the interaction between software and hardware (see the Supporting Information S1 for details). As the size of chemical systems to be simulated grows steadily larger, however, we argue that it is more important to reduce algorithmic complexity by searching for exploitable hidden structure.

If LOLCAT Method is able to take advantage of grouping reactions with common reactants as well as we believe it can, then LOLCAT Method may have a significant impact on what kinds of systems researchers are able to simulate. We hope the performance increase will help to transform computational biology into a more streamlined, interactive exercise.

## Supporting Information

Figure S1 (0.95 MB EPS)

Supporting Information S1 (0.31 MB PDF)
