# Supplementary material for: Reaction Factoring and Bipartite Update Graphs Accelerate the Gillespie Algorithm for Large-Scale Biochemical Systems
Source: PLoS One. 2010 Jan 6;5(1):e8125. doi: 10.1371/journal.pone.0008125 (PMC2798956; doi:10.1371/journal.pone.0008125)
Supplement: Supporting Information S1 — (0.31 MB PDF) [file pone.0008125.s002.pdf]

Supporting Information: Accelerating the Gillespie algorithm for large-scale biochemical systems

Sagar Indurkhya, Jacob Beal

November 5, 2009

1 Low-Level Optimizations

In the design of complex algorithms we generally eschew discussion of low-level optimizations because they often result in constant factor improvements that disappear when we examine asymptotic bounds. However, here we are concerned with both the design and implementation of the Gillespie Algorithm, so the optimizations that result in significant constant factor performance increases deserve mention. The depth of the topics we discuss varies, and it is important to note that these suggestions are only heuristic in nature based on our experiences and will almost certainly become out of date as computer processor and memory architectures develop; indeed the best suggestion we can give is to employ a programmer who has depth and breadth in current and state of the art code optimization. We present the following optimization strategies because it is also important for designers of algorithms to understand the actual cost of different types of computation, which is non-trivial when dealing with modern microprocessors.

Always use an optimizing compiler. The transition from a compiler that performs mediocre optimization to a compiler that targets specific instruction set extensions and hardware can result in dramatic runtime performance improvements. Many modern optimizing compilers offer services such as profile guided optimization that allows one to execute code on specified input data and then optimize the program under the assumption that similar data will be used at runtime.

Write code that avoids branch mispredictions. In our implementation of LOLCAT Method, the precompiler actually passes certain flags to the compiler when LOLCAT Method is being compiled, so that, for example, the `Update-Cloud-With-Primary` function's implementation actually has the full traversal up the main tree expanded in code (loop unrolling) to avoid the overhead of small loops. Other such optimizations are present throughout our implementation of LOLCAT Method.

Take advantage of numerically oriented instruction set extensions. LOLCAT Method, and more importantly the random number generator used were able to explicitly capture certain SSE3 and SSE4 floating point optimizations via the optimizing compiler.

Pay attention to Cache and Memory performance. The key idea here is to (1) organize data structures such that the data that is needed is locally available when possible (i.e. the data is in the registers or cache), and (2) design data structures to fit the data into the lowest level of memory possible. For example, in our results we found that on the largest model evaluated, ODM and NRM were not even able to store their update dependency graphs in the eight gigabytes of main memory available, while LOLCAT Method's UDG was able to fit in the four megabytes of L3 cache available. The speed difference then is both noticeable and something to be concerned with.

2 Using Heaps instead of Trees

Although we have described clouds and the main tree in terms of trees, each is actually implemented using a d-ary max-heap data structure rather than a balanced tree due to cache efficiency issues. A d-ary max-heap is a tree stored in memory as a linear array, in which each node has d children and the value of each node is greater than or equal to the value of any of it's d child nodes. In general, the d children of node t are nodes $d(t-1)+2$ through $dt+1$, and the parent node of node t is $\lfloor \frac{t-2}{d} + 1 \rfloor$. The root of the heap is located at position 1 in the array.

The cache is the processor's onboard memory (generally L1, L2 and L3 Cache for current processors. Missing the cache often incurs an approximately 10^3 cycle penalty, and a page-fault incurs a 10^6 cycle penalty. We use d-ary max-heaps (as opposed to a binary max-heap) because they give the heap a smaller height. This means that updating a heap, which requires traversing the nodes upwards from a leaf to the root, takes fewer operations. However, when we descend down a heap, we perform a number of comparisons; if the heap has height h, then we must perform $O(h(d-1)) = O(hd)$ comparison operations, since a linear search is performed over the d child nodes when descending down a node. As the heap grows larger in size and h increases, jumping down levels of the heap is likely to incur a cache miss, so up to a point it is faster to perform more comparisons per level in order to keep the number of levels smaller.

It should be noted that on modern processors from Intel and AMD, SSE instructions allow for the vectorization of floating point operations. If we instruct the processor to always predict the branching backwards while searching through a linear array, then if we have to search over d elements we incur only a single branch-misprediction. We found that $d = 64$ actually made each descent stage slightly faster than if we had used $d = 2$, while decreasing the height of the tree by a factor of $\log_2 64 = 6$.

3 Optimized Interpreter

One way in which our implementation of LOLCAT embraces low-level optimization is through compilation for an optimized interpreter. Bytecode for the optimized interpreter is emitted during the construction of clouds.

3.1 The Optimized Interpreter

In practice, the Gillespie Algorithm often requires a simulation program to compute tens of millions of iterations. LOLCAT Method uses a number of complex self-modifying data structures to take advantage of the ideas of grouping reactions by common reactants and using a sparse update dependency graph. The Optimized Interpreter (OI) is a carefully designed program that reads precompiled bytecode—a language of atomic instructions that instruct the OI what operations to perform on the data structures. In the case of the Optimized Interpreter, each atomic instruction is represented by a series of 32 bit integers[1].

The OI is guaranteed to always be in a state such that it will correctly interpret the bytecode it is about to execute (the bytecode can be interpreted differently based on the state of the OI), effectively eschewing any form of error detection or safety checks in favor of performance gain. This guarantee relies on an understanding that the bytecode generator will generate perfectly correct bytecode for the OI to consume.

Most of the instructions in the bytecode language are functions that update different parts of the data structures used or that set or examine set special states stored by the OI, such as the *CURRENT-CLOUD*.

Each reaction maps to a set of function calls (and the arguments to pass to these functions) that are stored sequentially in an array of integers, which we call the *reaction tape* (after the "tape" of a Turing machine). Each species also maps to a number of function calls (and the arguments to pass to these functions), and these are also stored sequentially in a separate array, which we call the *species tape*. Generally when reaction r^* is executed by the simulation, the simulation initiates the OI at the beginning of r^*'s section in the reaction tape. Here, each species that changes as a result of executing r^* is listed along with the amount by which that species will change, and the OI jumps to the appropriate position in the species tape for each species to execute the function calls that correctly update the data structures. See Figure 1 for further details and clarification. The reaction tape and species tape are both precompiled by the preprocessor and passed on to LOLCAT Method.

There are six base instructions used by the optimized interpreter:

1. `Update-Single-Reaction-In-SuperCache(n)`: Recalculates the propensity of the nth reaction in the *SuperCache*, and updates S_{SC}, for reactions where the reactants are different.

[1] Atomic instructions that are function calls are generally followed by values to pass to the function. Using an integer format lets each atom act as either a operation or a data value to pass on. This greatly increases the flexibility of what the OI can do.

2. `Update-Doublet-Reaction-In-SuperCache(n)`: Recalculates the propensity of the nth reaction in the *SuperCache*, and updates S_{SC}, for reactions where the reactants are the same.

3. `Set-Cloud(n)`: Set CUR-CLOUD to the nth cloud, then set CUR-CLOUD-TEMP to 0. The CUR-CLOUD-TEMP is an internal variable used to carry the sum of the operations that occur on a particular cloud during a given iteration. It should be equal to 0 before and after each iteration of the simulation.

4. `Update-Singlet-Reaction-In-Cloud(n, delta)`: Updates the CUR-CLOUD with the propensity of the nth singlet reaction in its primary tree. This instruction is an *internal operation*–an operation that mutates the data in the primary tree and sub-trees of a cloud.

5. `Update-Doublet-Reaction-In-Cloud(n, delta)`: Updates the CUR-CLOUD with the propensity of the nth doublet reaction in its primary tree. This instruction is an internal operation.

6. `Update-SubTree-In-Cloud(n, delta)`: Updates the CUR-CLOUD with the propensity of its nth sub-tree. This instruction is an internal operation.

7. `Update-Cloud-With-Primary(n)`: Updates the main tree with the new propensity of the CUR-CLOUD. Used after internal operations are performed on the CUR-CLOUD.

The above instructions can be considered first-order in that all of them are necessary to manipulate the data structures. The virtual machine can be trivially expanded to include more optimized instructions. For example, some sequences of first-order instructions appear so frequently that they can be handled by specialized instructions (and indeed these are present in our implementation of LOLCAT Method):

1. `Set-And-Update-Cloud-One-Pass(n, delta)`: Set CUR-CLOUD to the nth cloud, then updates the main tree with the new propensity of the CUR-CLOUD. Assumes no internal operations were performed on the cloud.

2. `Set-Cloud-Update-Reaction-No-Primary-Update-Cloud(`n_1`, `n_2`, delta)`: Set the CUR-CLOUD variable to the ${n_1}^{th}$ cloud, and update the ${n_2}^{th}$ reaction in the CUR-CLOUD's primary tree. Updates the cloud structure.

These instructions compress several of the above instructions. This has two advantages: first it allows us to compress the size of the dependency graph, and second it allows for micro-level optimizations to be performed in a very clean and elegant fashion.

Another example of how to extend the virtual machine is to add an instruction like *Record-Concentration*. This instruction is placed in the section of a species in the Species-Tape, and will automatically record the time and concentration of the desired species if and only if that species concentration

changes. This allows for extremely efficient exact recording[2] of species whose concentration changes rarely and whose concentration is critical to watch.

4 Modelling Systems with Dynamic Volume

We describe here a simple modification to LOLCAT Method that allows it to simulate variable volume systems. We create two sets of reactions: $R_{0,1}$ (the set of zero and first order reactions), and R_{2+} (the set of 2nd order and higher reactions). The propensities of the reactions in $R_{0,1}$ do not depend on the volume, whereas the reactions in R_{2+} do. Create two sets of all of the appropriate data structures (*SuperCache*, *Main Tree*, *Clouds*), for $R_{0,1}$ and R_{2+} respectively, and let them share a common Update Dependency Graph and Optimized Interpreter (so that a change in one set affects both sets of reactions appropriately).

The reaction rate of all reactions in R_{2+} are linearly proportional to the volume of the system being simulated, which means we can factor out the volume of the system from every second or higher order operation. Let us create all the data structures of R_{2+} using a volume of 1 (these reaction propensities will be referred to as normalized reaction propensities). Let $S_{R_{2+}}$ be the sum of the normalized reaction propensities in R_{2+}, $S_{R_{0,1}}$ be the sum of the reaction propensities of all reactions in $S_{R_{0,1}}$, and $V(t)$ be the volume of the system at time t.

Then we can modify the first step of LOLCAT Method to choose which Main Tree to descend down:

$$P(\text{Descend Down } R_{0,1} \text{ Main Tree}) = 1 - \frac{V(t)S_{R_{2+}}}{S_{R_{0,1}} + V(t)S_{R_{2+}}}$$

Everything else in LOLCAT Method can stay the same. Note that the complexity of the algorithm does not increase at all, because all we are doing is an additional $O(1)$ time operation each iteration (to choose which Main Tree to descend down).

[2]By exact recording we mean that we have will know what the concentration of that species was at all points in time during the simulation

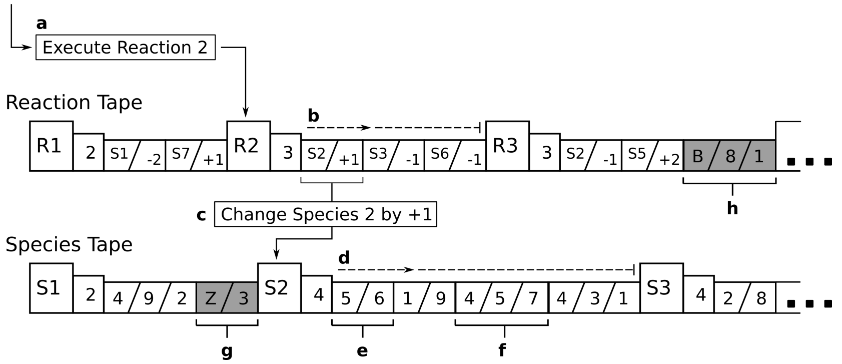

Figure 1: **Execution and update of a reaction:** (**a**) The simulation has chosen to execute reaction 2 (R_2), and the Optimized Interpreter (OI) jumps to the beginning of the R_2 section of the Reaction Tape. (**b**) Here the OI will change the concentrations of the 3 species S_2, S_3 and S_6 by $+1$, -1 and -1 respectively, and perform the necessary updates on the data structures. (**c**) Demonstrates the OI changing the concentration of species S_2 by $+1$ and jumping to the beginning of the S_2 section of the Species Tape. (**d**) Here the OI will execute the next four function calls on the Species Tape before jumping back to where it left the Reaction Tape. Examples of function calls include: (**e**) set the current cloud to cloud 6, and (**f**) set the current cloud to 5, update reaction 7 in this cloud, and update the current cloud. We also display how specialized function calls can be placed to trigger when a particular species changes in concentration (**g**) or a particular reaction occurs (**h**).
